# Influence of steeping duration, drying temperature, and duration on the chemical composition of sorghum starch

**DOI:** 10.1002/fsn3.562

**Published:** 2017-12-15

**Authors:** Lukumon A. Odunmbaku, Sunday S. Sobowale, Monilola K. Adenekan, Taiwo Oloyede, Janet A. Adebiyi, Oluwafemi A. Adebo

**Affiliations:** ^1^ Department of Food Technology Moshood Abiola Polytechnic Abeokuta Ogun State Nigeria; ^2^ Department of Biotechnology and Food Technology Faculty of Science University of Johannesburg Johannesburg Gauteng South Africa

**Keywords:** amylose, chemical properties, optimization, sorghum, starch

## Abstract

The quest for high‐quality starch that would meet the needs of manufacturers is ever increasing. This study investigated the effect of steeping duration, drying temperature, and duration on the chemical properties of sorghum starch, to possibly alter the characteristics of sorghum starch for food applications. Steeping duration, drying temperature, and drying time of starch isolation were optimized using a central composite design and nine parameters including pH, amylose content, moisture, protein, ash, crude fiber, fat, carbohydrate, and total energy determined. Results obtained showed that most of the parameters were majorly influenced by steeping and drying duration. Steeping duration significantly (*p* < .05) increased the moisture, protein, and ash content of the sorghum with a corresponding decrease in pH values. The obtained experimental and predicted values of the investigated parameters were similar, with statistical indices indicating the relative validity of the generated models [absolute average deviation (AAD between 0 and 0.20), bias factor (*B*_*f*_, 1–1.02), and accuracy factor (*A*_*f*_, 1–1.21)]. The varying values of the parameters obtained indicates the potential use of the sorghum starches as thickeners, starch substitutes, and for other desired roles in food processing.

## 1 INTRODUCTION

Sorghum (*Sorghum bicolor*) is a drought‐resistant grass specie, majorly cultivated for its grain use. It is the 5th most important cereal crop in the world after rice, wheat, maize, barley (Taylor & Emmambux, 2008), an important cereal crop and major source of food for millions of people in Africa (Adebo et al., 2017; Taylor, Schober, & Bean, 2006). Data from the Food and Agriculture Organization statistics (FAOSTAT) indicate that the world production of sorghum is 68,938,587 tonnes, with Nigeria contributing 6,741,100 tonnes to this (FAOSTAT, 2017). The ability of sorghum to grow and propagate under harsh conditions and its photosynthetic efficiency strongly position its increased utilization for both food and nonfood products (Mutsiya et al., 2003). One viable way of utilizing sorghum and creating more economic value would be through starch production from this versatile cereal grain.

Starch is a carbohydrate consisting of a large number of glucose units joined by glycosidic bond (Bertoft & Nilsson, 2017). It is a major ingredient in foods, used in a variety of food products as a raw material, food additive, and as fat substitutes. Depending on the desired application, the functional, physical, and chemical structure of starches can be altered through modification. Physically achieving this (through steeping) is not only cost effective, safe, relatively easy, but can also significantly improve the quality of sorghum starch and reduce antinutrients (Claver, Zhang, Li, Zhu, & Zhou, 2010). Sorghum starch has been reported to have similar properties as that of corn and a potentially good source of raw materials for a wide range of uses (Beta, Corke, & Taylor, 2000; Beta, Obilana, & Corke, 2001; Emmambux & Taylor, 2013; Singh, Sodhi, & Singh, 2010; Srichuwong et al., 2017).

Subsequent characterization and investigation of the properties of such starches is particularly important prior to industrial and food applications. Furthermore, the current demand for starches have been met by relatively few crops (Adeboye & Emmambux, 2016), necessitating the need to explore other readily available sources like sorghum. Sequel to this, this study was therefore carried out to investigate the effect of steeping duration, drying temperature, and duration on the chemical properties of sorghum starch.

## 2 MATERIAL AND METHODS

### 2.1 Raw material and sample preparation

Sorghum (*Sorghum bicolor*) grains used for the study were purchased from Lafenwa market in Abeokuta (7.15°N, 3.35°E), Ogun State, Nigeria. The grains were subsequently sorted and cleaned. Damaged grains, stones, and other extraneous materials removed and discarded.

### 2.2 Optimization of parameters

Using a central composite design (CCD) on MATLAB statistical software (MathWorksInc, Massachusetts, USA), experimental sets were obtained to investigate the influence of three independent variables, steeping duration (*X*_1_), drying temperature (*X*_2_), and drying duration (*X*_3_). The three‐factor design gave a total of 15 experiments (Table 1). Nine (9) responses namely, pH (*Y*_1_), amylose content (*Y*_2_), moisture content (*Y*_3_), protein content (*Y*_4_), ash content (*Y*_5_), crude fiber (*Y*_6_), fat content (*Y*_7_), carbohydrate content (*Y*_8_), and total energy (*Y*_9_) were evaluated. The mathematical model describing the relationship between the independent variables in terms of their linear, quadratic and interaction effects is described by a second‐order polynomial equation, presented in Equation (1).

(1) $$\begin{array}{cc} Y= & \;\alpha_{0}+\alpha_{1}x_{1}+\alpha_{2}x_{2}+\alpha_{3}x_{3}+\alpha_{11}x_{1^{2}}+\alpha_{22}x_{2^{2}}+\alpha_{33}x_{3^{3}}+\alpha_{12}x_{1}x_{2}\\ & +\alpha_{13}x_{1}x_{3}+\alpha_{23}x_{2}x_{3}+\ldots \end{array}$$

where α_o_, α_1_–α_3_, α_11_–α_33_, and α_12_–α_13_ are the equation regression coefficients for intercept, linear, quadratic, and interaction coefficient, respectively, *x*_1_–*x*_3_ are coded independent variables.

**Table 1:** Coded and real values for the CCD design

Experimental (Exp) runs	Coded values			Real values		
	*X*_1_	*X*_2_	*X*_3_	*X*_1_ (h)	*X*_2_ (°C)	*X*_3_ (min)
1	−1	−1	−1	12	60	30
2	1	−1	−1	48	60	30
3	−1	1	−1	12	70	30
4	1	1	−1	48	70	30
5	−1	−1	1	12	60	180
6	1	−1	1	48	60	180
7	−1	1	1	12	70	180
8	1	1	1	48	70	180
9	−1	0	0	12	65	105
10	1	0	0	48	65	105
11	0	−1	0	30	60	105
12	0	1	0	30	70	105
13	0	0	−1	30	65	30
14	0	0	1	30	65	180
15	0	0	0	30	65	105

*X*_1_, steeping duration; *X*_2_, drying temperature; *X*_3_, drying time.

### 2.3 Sorghum starch production

**Figure 1:**
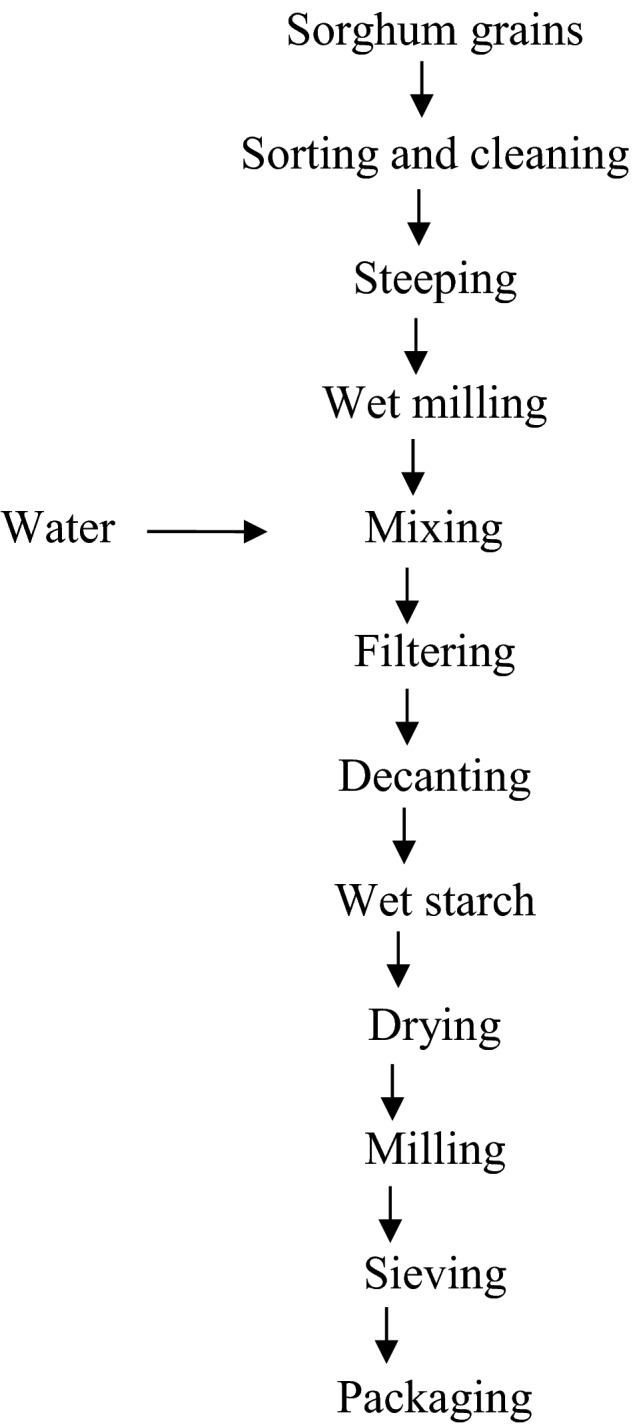
Flowchart for the production of starch from sorghum (Adapted from Singh et al., 2009)

The cleaned sorghum grains were steeped for different times (Table 1) using the procedure of Singh, Sodhi, and Singh (2009) with slight modification (Figure 1). The sorghum grains were then wet‐milled into a smooth paste and mixed with clean water (1:5, w/v), filtered through muslin cloth and allowed to settle. The supernatant was decanted, the sediment dewatered with cheese‐clothe and the starch washed three times with water. The starch cake was broken, spread thinly on trays and dried in a hot air oven (Gallemkamp Scientific, UK) using the time and temperature combinations presented in Table 1. The obtained samples at each of the experimental runs were subsequently sieved (through a 100 μm sifter), packaged in high‐density polyethylene bags and stored at 4°C prior to analysis.

### 2.4 Percentage starch yield determination

The percentage yield of the starch was determined according to the method described by Akanbi, Nazamid, and Adebowale (2009). Starch yield (%) = (Weight of starch/weight of sorghum grain) × 100.

### 2.5 pH

2 g of the sample was dispersed in 20 ml of distilled water. The pH was, thereafter, measured using a pH meter (WPH CD70).

### 2.6 Determination of amylose content

Amylose content was determined using the methods of Williams, Kuzina, and Hlynka (1970) and Udachan, Sahoo, and Hend (2012) with optical density measurement (Spectrumlab 22pc, Rinch Industrial, China) at 620 nm.

### 2.7 Proximate composition and total energy value

Moisture content of the samples was determined according to the method described by AOAC (2004). The samples were weighed into preweighed flasks and dried in the oven (Gallemkamp Scientific, UK) at 105°C until constant mass. Percentage differences between the initial and final weight of the samples were recorded as percentage moisture content. Other proximate components including crude protein (Kjeldahl method), ash content, crude fiber, and crude fat (Soxhlet extraction) were, respectively, determined using methods 990.03, 923.03 (32.1.05), 978.10, and 920.39 (A) of AOAC (2006). Total carbohydrate was determined by difference (AOAC, 2006), whereas the total energy was calculated using the Atwater factors [Energy value (kcal) = (% Protein × 4 + % Carbohydrate × 4 + % Fat × 9.0)] (FAO, 2003).

### 2.8 Statistical analysis

All analyses were done in triplicate and results presented represent the average of triplicate determinations, expressed as mean and standard deviation. The data obtained were analyzed by analysis of variance (ANOVA) using SPSS Statistics 22 software (IBM, USA). Significant *F* tests at (*p* < .05) levels of probability are reported. Statistical models were generated using Minitab 16 statistical software (Minitab Lt. Coventry, UK) and were also used to execute ANOVA on the models at 5% confidence level. To validate the model equations obtained, the average absolute deviation (AAD), bias factor (*B*_*f*_), and accuracy factor (*A*_*f*_) were calculated using Equations (2), (3), (4). The coefficient of determination (*R* ^2^), was also obtained to compare the experimental and calculated values given by the models.

(2) $$\text{AAD}=\;\frac{\left[\sum_{i=1}^{N}\left(\frac{\left|Y_{i,\exp}-\;Y_{i,\;\mathrm{cal}}\right|}{Y_{i,\exp}}\right)\right]}{N}$$

(3) $$B_{f}=10^{\frac{1}{N}}\sum_{i=1}^{N}\log\left(\frac{Y_{i,\;\mathrm{cal}}}{Y_{i,\exp}}\right)$$

(4) $$A_{f}=10^{\frac{1}{N}}\sum_{i=1}^{N}\left|\log\left(\frac{Y_{i,\;\mathrm{cal}}}{Y_{i,\exp}}\right)\right|$$

## 3 RESULTS AND DISCUSSION

### 3.1 Starch yield

**Figure 2:**
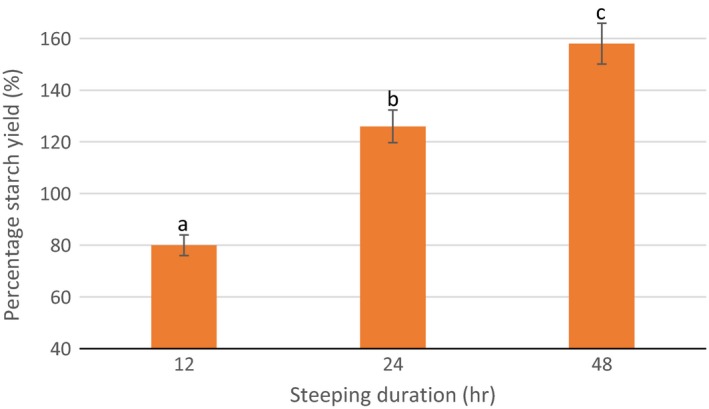
Effect of steeping duration on starch yield

The starch yield as affected by the steeping duration was evaluated immediately after steeping of the sorghum grains. As shown in Figure 2, the starch yield was observed to significantly (*p* < .05) increase with steeping duration (12, 24, and 48 hr). This could mean that as steeping duration increased, there was more degradation of large molecular structures (Adebiyi, Obadina, Mulaba‐Bafubiandi, Adebo, & Kayitesi, 2016; Adebo et al., 2017), possibly leading to an increase in starch particle size which contributed to the increase in starch yields. This might also be attributed to the dissolution or breakage of bonds between the protein and starch leading to better starch separation.

### 3.2 Statistical models and validation

This study investigated the effects of independent process variables [steeping duration (*X*_1_), drying temperature (*X*_2_), and drying time (*X*_3_)] on the production of starch from sorghum. Parameters determined were pH (*Y*_1_), amylose content (*Y*_2_), moisture content (*Y*_3_), protein content (*Y*_4_), ash content (*Y*_5_), crude fiber (*Y*_6_), fat content (*Y*_7_), carbohydrate content (*Y*_8_), and total energy (*Y*_9_) and the different models representing each provided in Equations (5), (6), (7), (8), (9), (10), (11), (12), (13).

(5) $$\begin{array}{cc} Y_{1}\;=\; & 3.82489-0.906x_{1}-0.056x_{2}-0.046x_{3}+0.41889x_{1^{2}}\\ & +0.06889x_{2^{2}}+0.21889x_{3^{2}}-0.095x_{1}x_{2}-0.02x_{1}x_{3}+0.005x_{2}x_{3} \end{array}$$

(6) $$\begin{array}{cc} Y_{2} & \;=\;27.3242-0.886x_{1}+1.124x_{2}-0.295x_{3}-0.3578x_{1^{2}}\\ & \;-0.0478x_{2^{2}}-0.2828x_{3^{2}}-0.4050x_{1}x_{2}-1.8875x_{1}x_{3}-1.8425x_{2}x_{3} \end{array}$$

(7) $$\begin{array}{cc} Y_{3}\;=\; & 13.4453-0.333x_{1}-0.3520x_{2}-0.566x_{3}-0.2317x_{1^{2}}\\ & -0.3367x_{2^{2}}+0.2733x_{3^{2}}-0.2425x_{1}x_{2}-0.1275x_{1}x_{3}+0.35x_{2}x_{3} \end{array}$$

(8) $$\begin{array}{cc} Y_{4}\;=\; & 5.37667+0.881x_{1}+0.099x_{2}+0.09x_{3}+0.76167x_{1^{2}}-0.12833x_{2^{2}}\\ & -0.11333x_{3^{2}}+0.10875x_{1}x_{2}+0.11875x_{1}x_{3}+0.12125x_{2}x_{3} \end{array}$$

(9) $$\begin{array}{cc} Y_{5}\;=\; & 0.52667+0.096x_{1}+0.026x_{2}+0.032x_{3}-0.01333x_{1^{2}}\\ & +0.02667x_{2^{2}}+0.01667x_{3^{2}}-0.025x_{1}x_{2}-0.02x_{1}x_{3}-0.0175x_{2}x_{3} \end{array}$$

(10) $$\begin{array}{cc} Y_{6}\;=\; & 1.34978+0.027x_{1}-0.03x_{2}+0.002x_{3}+0.18278x_{1^{2}}\\ & +0.18778x_{2^{2}}-0.14222x_{3^{2}}-0.00375x_{1}x_{2}-0.00625x_{1}x_{3}\\ & +0.00375x_{2}x_{3} \end{array}$$

(11) $$\begin{array}{cc} Y_{7}\;=\; & 2.30044+0.009x_{1}-0.462x_{2}+0.611x_{3}-0.25056x_{1^{2}}-0.03556x_{2^{2}}\\ & +0.69944x_{3^{2}}-0.18625x_{1}x_{2}+0.27125x_{1}x_{3}-0.13625x_{2}x_{3} \end{array}$$

(12) $$\begin{array}{cc} Y_{8}\;=\; & 76.8916-0.679x_{1}+0.776x_{2}-0.183x_{3}-0.3294x_{1^{2}}+0.1556x_{2^{2}}\\ & -0.6194x_{3^{2}}+0.2987x_{1}x_{2}-0.2763x_{1}x_{3}-0.3313x_{2}x_{3} \end{array}$$

(13) $$\begin{array}{cc} Y_{9}\;=\; & 349.777+0.889x_{1}-0.658x_{2}+5.127x_{3}-0.526x_{1^{2}}-0.211x_{2^{2}}\\ & +3.364x_{3^{2}}-0.046x_{1}x_{2}+1.811x_{1}x_{3}-2.066x_{2}x_{3} \end{array}$$

All calculated *R* ^2^ values in this study were above 80, except for that of crude fiber (*Y*_6_), fat content (*Y*_7_), and total energy (*Y*_9_) (Table 2). *R* ^2^ values should be at about 80% to have a good fit of the model and the closer it is to 100%, the better the empirical model fits the actual data (Adebo et al., 2017; Sobowale, Adebiyi, & Adebo, 2017). Nevertheless, other parameters of predictive models in biological systems that measure the relative deviation from the observed (experimental) and predicted (calculated) parameters were determined and acceptable results (Table 2) still allow for a valid model and interpretation. As observed, the relative closeness of the bias factor (*B*_*f*_) and accuracy factor (*A*_*f*_) to unity (1) and that of average absolute deviation (AAD) to zero indicates reasonable agreements between the predicted and observed parameters (Adebo et al., 2017; Sobowale et al., 2017).

**Table 2:** Coefficient of regression, *R* ^2^, AAD, *B*_*f*_, and *A*_*f*_ values for the mathematical models of the responses

Coefficient	*Y*_1_	*Y*_2_	*Y*_3_	*Y*_4_	*Y*_5_	*Y*_6_	*Y*_7_	*Y*_8_	*Y*_9_
α_0_	3.82489	27.3242	13.4453	5.37667	0.52667	1.34978	2.30044	76.8916	349.777
α_1_	–0.906^a^	–0.886	–0.333	0.881^a^	0.096^a^	0.027	0.009	–0.679^a^	0.889
α_2_	–0.056	1.124^a^	–0.352	0.099	0.026	–0.03	–0.462	0.776^a^	–0.658
α_3_	–0.046	0.295	–0.566^a^	0.09	0.032^a^	0.002	0.611	–0.183	5.127^a^
α_11_	0.41889^a^	–0.3578	–0.2317	0.76167^a^	–0.01333	0.18278	–0.25056	–0.3294	–0.526
α_22_	0.06889	–0.0478	–0.3367	–0.12833	0.02667	0.18778	–0.03556	0.1556	–0.211
α_33_	0.21889	–0.2828	0.2733	–0.11333	0.01667	–0.14222	0.69944	–0.6194	3.364
α_12_	–0.095	–0.4050	–0.2425	0.10875	0.025	–0.00375	–0.18625	0.2987	–0.046
α_13_	–0.02	–1.8875^a^	–0.1275	0.11875	0.02	–0.00625	0.27125	–0.2763	1.811
α_23_	0.005	–1.8425^a^	0.35	0.12125	–0.0175	0.00375	–0.13625	–0.3313	–2.066
*R* ^2^ (%)	98.95	90.91	85.13	97.37	95.52	48.72	67.46	83.97	63.77
AAD	0.02	0.02	0.02	0.02	0.03	0.08	0.20	0.00	0.01
*B*_*f*_	1.00	1.00	1.00	1.00	1.00	1.00	1.02	1.00	1.00
*A*_*f*_	1.02	1.02	1.02	1.02	1.03	1.08	1.21	1.00	1.01

AAD, average absolute deviation; *B*_*f*_, bias factor; *A*_*f*_ *,* accuracy factor.

*Y*_1_—pH, *Y*_2_—amylose content, *Y*_3_—moisture content, *Y*_4_—protein content, *Y*_5_—ash content, *Y*_6_—crude fiber, *Y*_7_—fat content, *Y*_8_—carbohydrate content, and *Y*_9_—total energy. α_0_, α_1_–α_3_, α_11_–α_33_,and α_12_–α_13_ are the equation regression coefficients for intercept, linear, quadratic, and interaction coefficient, respectively, *x*_1_–*x*_3_ are coded independent variables. *R* ^2^, coefficient of determination.

a Significant at *p *≤* *.05.

### 3.3 pH and amylose content

**Figure 3:**
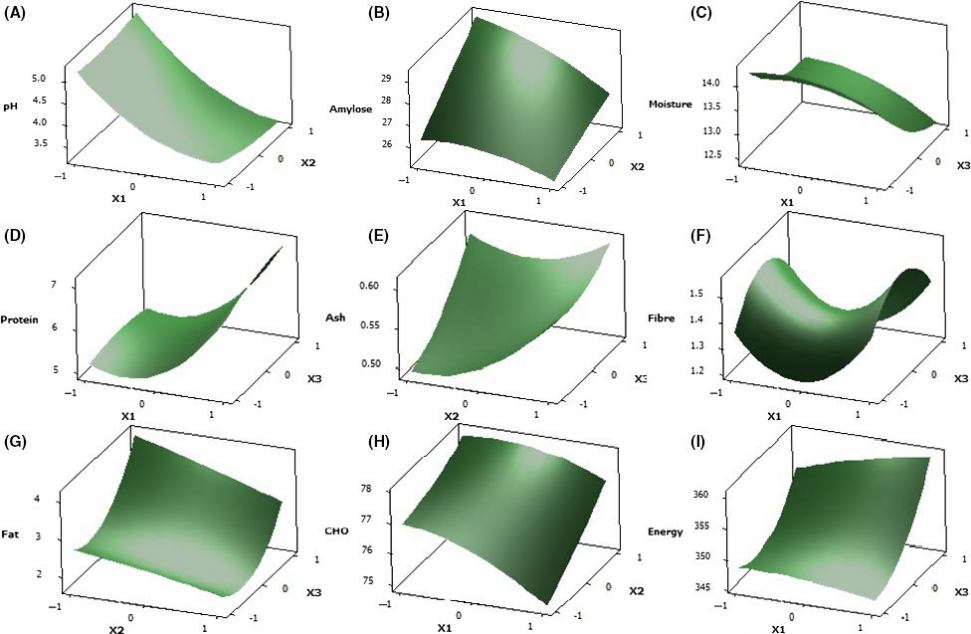
Surface plots of the responses investigated (A)*Y*_1_—pH, (B)*Y*_2_—amylose content, (C)*Y*_3_—moisture content, (D)*Y*_4_—protein content, (E)*Y*_5_—ash content, (F) *Y*_6_—crude fiber, (G)*Y*_7_—fat content, (H)*Y*_8_—carbohydrate content, and (I)*Y*_9_—total energy

It was observed that as the steeping duration increased, the pH value of the sorghum starch samples decreased (Table 3). This suggests increased hydrolysis and accelerated action of microorganisms leading to the recorded drop in the pH values. As observed from the regression coefficients of the pH model (*Y*_1_) (Table 2), only the linear (*X*_1_) and quadratic effect ($X_{1}^{2}$) of steeping duration had respective negative and positive significant effect (*p* < .05) on the pH of sorghum starch. The surface plots on Figure 3A also show that an increase in steeping duration would cause a decrease in pH. Both drying temperature and duration were observed not to have a pronounced or significant (*p* < .05) effect on the pH because all sample are still in the same medium.

**Table 3:** Chemical composition of sorghum starch

Variables			*Y*_1_		*Y*_2_ (%)		*Y*_3_ (%)		*Y*_4_ (%)		*Y*_5_ (%)		*Y*_6_ (%)		*Y*_7_ (%)		*Y*_8_ (%)		*Y*_9_ (kcal)	
*X*_1_ (h)	*X*_2_ (°C)	*X*_3_ (min)	Exp	Pred	Exp	Pred	Exp	Pred	Exp	Pred	Exp	Pred	Exp	Pred	Exp	Pred	Exp	Pred	Exp	Pred
12	60	30	5.40^gh^(0.00)	5.43	20.90^a^(0.03)	21.97	14.23^i^(0.00)	14.38	5.08^bc^(0.02)	5.18	0.42^a^(0.02)	0.43	1.51^e^(0.02)	1.57	2.80^g^(0.08)	2.42	75.96^f^(0.03)	75.88	349.36^e^(0.10)	346.75
48	60	30	3.80^d^(0.00)	3.85	25.09^d^(0.01)	24.78	14.71^j^(0.00)	14.46	6.48^f^(0.02)	6.48	0.55^fg^(0.01)	0.53	1.84^j^(0.02)	1.64	2.16^d^(0.09)	2.43	74.26^b^(0.99)	74.47	342.40^a^(0.08)	344.99
12	70	30	5.40^gh^(0.03)	5.50	29.00^i^(0.00)	28.71	13.80^h^(0.29)	13.46	5.07^bc^(0.01)	4.91	0.46^bc^(0.01)	0.47	1.62 ^fg^(0.21)	1.52	1.49^a^(0.10)	2.14	77.59 ^m^(0.27)	77.49	344.05^b^(0.15)	349.65
48	70	30	3.60^c^(0.14)	3.54	29.95^k^(0.07)	29.90	12.10^ab^(0.10)	12.57	6.49^f^(0.01)	6.66	0.68^j^(0.01)	0.67	1.40^d^(0.00)	1.58	1.50^ab^(0.10)	1.41	77.87^n^(0.10)	77.28	350.94^gh^(0.11)	347.72
12	60	180	5.30^g^(0.14)	5.37	29.94^k^(0.07)	30.02	13.24^ef^(0.00)	12.80	5.03^a^(0.02)	4.88	0.48^cd^(0.01)	0.49	1.74^hi^(0.02)	1.59	3.36^j^(1.06)	3.38	76.13 ^g^(2.59)	76.72	354.88^jk^(0.22)	357.51
48	60	180	3.80^d^(0.00)	3.71	24.96^k^(0.01)	25.28	12.00^a^(0.00)	12.37	6.49^f^(0.01)	6.66	0.68^j^(0.01)	0.67	1.51^e^(0.01)	1.64	5.20^k^(1.00)	4.47	74.11^a^(0.01)	74.22	369.20^n^(0.09)	363.00
12	70	180	5.50^h^(0.00)	5.46	29.05^ij^(0.07)	29.39	13.00^c^(0.00)	13.29	5.09^cd^(0.01)	5.10	0.44^ab^(0.01)	0.46	1.33^c^(0.02)	1.53	2.90^i^(0.09)	2.55	77.22^k^(0.03)	77.02	355.34^l^(0.13)	352.15
48	70	180	3.44^b^(0.07)	3.41	24.07^ij^(0.02)	23.03	12.00^a^(0.00)	11.88	7.40^h^(0.06)	7.32	0.75^k^(0.01)	0.74	1.62 ^fg^(0.01)	1.56	2.60^f^(0.08)	2.90	75.61^c^(0.37)	75.70	355.44^lm^(0.04)	357.46
12	65	105	5.30^g^(0.00)	5.15	29.05^ij^(0.07)	27.85	13.21^de^(0.02)	13.55	5.05^ab^(0.07)	5.26	0.46^bc^(0.04)	0.42	1.51^e^(0.00)	1.51	2.31^e^(1.06)	1.96	77.45 ^l^(0.09)	77.24	350.79^fg^(0.09)	348.36
48	65	105	3.20^a^(0.00)	3.34	25.01^cd^(0.01)	26.08	13.34^gh^(0.01)	12.88	7.27 ^g^(0.06)	7.02	0.56^gh^(0.01)	0.61	1.61^f^(0.01)	1.56	1.49^a^(1.00)	2.14	75.71^d^(0.05)	75.88	345.33^c^(0.08)	350.14
30	60	105	4.00^e^(0.00)	3.95	27.31^gh^(0.01)	26.15	13.29^ef^(0.01)	13.46	5.26^d^(0.01)	5.15	0.52^ef^(0.01)	0.53	1.40^d^(0.01)	1.57	1.91^c^(0.90)	2.73	77.10^jk^(0.62)	76.27	346.33^d^(0.11)	350.22
30	70	105	3.80^d^(0.00)	3.84	27.37^gh^(0.01)	28.40	13.05^cd^(0.07)	12.76	5.28^de^(0.01)	5.35	0.58^hi^(0.01)	0.58	1.73^h^(0.01)	1.51	2.32^e^(1.02)	1.80	77.03^j^(0.09)	77.82	350.12^f^(0.05)	348.91
30	65	30	4.20^f^(0.07)	4.09	27.17^g^(0.24)	26.75	14.31^j^(0.01)	14.28	5.28^de^(0.01)	5.17	0.50^de^(0.01)	0.51	1.14^a^(0.01)	1.21	2.85^h^(0.19)	2.39	75.90^e^(0.07)	76.46	350.37^f^(0.03)	348.01
30	65	180	3.90^de^(0.03)	4.00	27.04^e^(0.01)	27.34	13.25^ef^(0.01)	13.15	5.29^de^(0.01)	5.35	0.58^hi^(0.01)	0.58	1.33^c^(0.02)	1.21	2.85^h^(0.77)	3.61	76.68^h^(0.03)	76.09	353.53^i^(0.10)	358.27
*X*_1_ (h)	*X*_2_ (°C)	*X*_3_ (min)	Exp	Pred	Exp	Pred	Exp	Pred	Exp	Pred	Exp	Pred	Exp	Pred	Exp	Pred	Exp	Pred	Exp	Pred
30	65	105	3.80^d^(0.02)	3.82	27.07^ef^(1.85)	27.32	13.20^d^(0.01)	13.45	5.29^de^(0.01)	5.38	0.54 ^fg^(0.04)	0.53	1.24^b^(0.01)	1.35	2.90^i^(0.85)	2.30	76.82^i^(0.05)	76.89	354.54^j^(0.11)	349.78

*X*_1_—steeping duration; *X*_2_—drying temperature; *X*_3_—drying duration, *Y*_1_—pH, *Y*_2_—amylose content, *Y*_3_—moisture content, *Y*_4_—protein content, *Y*_5_—ash content, *Y*_6_—crude fiber, *Y*_7_—fat content, *Y*_8_—carbohydrate content, and *Y*_9_—total energy; Exp—experimental value; Pre—predicted value. Values in parentheses represent the standard deviation of triplicate measurements. Means with no common letters within a column significantly differ (*p* < .05).

Amylose is an important parameter and component of starches. They play major and significant role in pasting, gelatinization, swelling, gel firmness and viscosity, contributing to the strength, and behavior of the sorghum starch. The amylose content of the sorghum starches ranged from 20 to approximately 30% (Table 3), comparable with those earlier reported for sorghum starches (Gaffa et al., 2004; Olayinka, Adebowale, & Olu‐Owolabi, 2013; Singh et al., 2010; Sun, Han, Wang, & Xion, 2014; Udachan et al., 2012). The highest and lowest values obtained were at experimental run 1 and 4 (Table 1), respectively. These values suggest that steeping sorghum starch for a longer time and using a high temperature and short drying duration would significantly (*p* < .05) influence the amylose content. This is also in agreement with the studies of Claver et al. (2010) that suggested generation of more amylose‐based structure during soaking, but leaching and solubilization of same with heat (Zhu, 2014). Heat might have also influenced rearrangement of the starch molecules, thereby contributing to reduction in the amylose contents. Gelatinization at relatively higher temperatures (Singh et al., 2009, 2010) and possible formation of complexes and intermolecular interactions (Sun et al., 2014) might have equally contributed to the observed changes in amylose content. During the steeping process of cereals, complex structures, and nutrients are usually degraded by endogenous microorganisms (Adebiyi et al., 2016; Adebo et al., 2017) and can also be attributed to the trend of amylose content observed in this study. Considering the coefficients of regression of the model, only the linear effect of drying temperature (*X*_2_) and the quadratic interaction effects of steeping and drying duration (*X*_1_ *X*_3_) and drying temperature and duration (*X*_2_ *X*_3_) significantly (*p* < .05) influenced the amylose content. This is also reflected in the surface plot presented in Figure 3B, in which the parameter was observed to increase with increasing steeping duration, drying temperature and reduce with increasing drying time.

### 3.4 Proximate composition and energy value

The proximate composition of the sorghum starch samples is presented in Table 3. The results obtained were relatively comparable with the proximate composition of sorghum starch reported by earlier authors (Udachan et al., 2012; Zhu, 2014). These contents ranged between 12% and 14.71% (moisture), 5.03%–7.4% (protein), 0.42%–0.5% (ash), 1.14%–1.84% (fiber), 1.49%–5.2% (fat), 74.11%–77.87% (carbohydrate), and 342.4%–369 kcal (energy values) (Table 3). Considering the regression coefficients (Table 2), only the linear negative effect of drying duration (*X*_3_) was significant (*p* < .05) on the moisture content of the sorghum starch, suggesting that an increase in drying duration yield a decrease in moisture content and vice versa (Figure 3C). Such decrease would likely give the product a better keeping quality thus prolonging its shelf life. As anticipated, only the positive linear and quadratic effects of steeping duration (*X*_1_and $X_{1}^{2}$, respectively) had significant (*p* < .05) effect on protein content (Table 2). The surface plot (Figure 3D) and results obtained (Table 3) further indicates that increase in the duration of steeping increases protein. This trend can be due to the depolymerization of the protein molecules and accumulation of amino acids with steeping (Adebiyi et al., 2016).

It was further observed that the linear effects of steeping and drying duration (*X*_1_ and *X*_3_) had significant (*p* < .05) effect on the ash content, steeping duration, and drying temperature (*X*_1_ and *X*_2_) on carbohydrate and only that of drying duration (*X*_3_) on the energy values (Table 2). While increase in the ash contents could be due to losses of dry mater (Uvere, Onyekwere, & Ngoddy, 2010), higher carbohydrate contents could be attributed to the conversion and solubilization of high molecular weight carbohydrates to simpler ones. Increases in these contents are desirable in starches, especially for use in the formulation of pastries, bakery products, use in gluten free products, and other food applications. None of the linear, quadratic or interaction effects had a significant effect on the crude fiber and fat content of the sorghum starch samples (Table 2). The surface plot of both parameters nonetheless shows the influence of the variables on them. The role of fat/lipids in starches and cereals have been acknowledged by other authors as being able to influence the swelling and pasting properties (Goering, Jackson, & De Haas, 1975; Thongngam & Chanapamokkhot, 2007).

## 4 CONCLUSION

Sorghum is an inexpensive, readily available source of food and an alternative starch source. Results obtained in this study suggest the susceptibility of the investigated sorghum starch parameters to steeping duration, drying temperature, and duration. Changes in the various parameters determined may be attributed to structural changes, molecular disruption and disintegration, probable modifications in particle sizes, breakage of bonds, and intermolecular interactions. The results showed that sorghum starch exhibited interesting properties, essential for food formulations, potential functional ingredient, and for possible use in other industrial applications where certain characteristics are desired. The optimal processing conditions for the processing of sorghum starch in this study was steeping time of 48 hr, drying temperature of 70°C and a drying duration of 180 min. At these conditions, desirable values for all the investigated parameters were obtained. Nonetheless, since the applications of starch are majorly guided by their physicochemical properties, subsequent use and purpose would influence the choice of sorghum starch obtained in this study. Further characterization of these starches is still needed to further understand their similarities and differences, for potential use in the food industry.

## CONFLICT OF INTEREST

None declared.
